# Comparative effectiveness of debridement strategies for chronic lower-extremity wounds: a systematic review and network meta-analysis

**DOI:** 10.3389/fmed.2026.1852029

**Published:** 2026-05-21

**Authors:** Guorui Li, Xiaoyun Zhang, Suiyi Liu, Lin Chen, Sha Liu

**Affiliations:** 1 The Burn and Plastic Surgery Department of the 944th Hospital of the Joint Support Force, Jiuquan, Gansu, China; 2 Shandong Provincial Engineering Research Center of Novel Pharmaceutical Excipients and Controlled Release Preparations, College of Health and Medicine, Dezhou University, Dezhou, Shandong, China; 3 Department of Plastic and Aesthetic Surgery, Affiliated Hospital of Shaanxi University of Chinese Medicine, Xianyang, Shaanxi, China

**Keywords:** biological debridement, chronic lower-extremity wounds, debridement, enzymatic debridement, network meta-analysis, wound healing

## Abstract

**Objective:**

This study aimed to compare debridement strategies for chronic lower-extremity wounds using a systematic review and Bayesian network meta-analysis.

**Methods:**

This study followed the PRISMA-NMA guidelines. PubMed, Embase, Web of Science, the Cochrane Library, CNKI, Wanfang, and VIP were searched from January 1990 to February 2026. Randomized controlled trials involving venous leg ulcers, arterial or ischemic ulcers, mixed arterial–venous ulcers, or diabetic foot ulcers were included. The outcomes included wound healing, complete debridement, pain score, procedure time, and time to healing. Pairwise meta-analysis and Bayesian network meta-analysis were performed. Surface under the cumulative ranking curve (SUCRA) rankings were interpreted according to the clinical direction of each outcome.

**Results:**

A total of 25 randomized controlled trials were included. For wound healing, biological debridement (BIO) had the highest ranking probability (SUCRA = 99.5%), followed by enzymatic debridement (ENZ) (83.1%) and mechanical debridement (MECH) (65.9%). For complete debridement, biological debridement ranked first (95.7%), followed by enzymatic debridement (70.9%), autolytic debridement (AUTO) (32.2%), and standard care (SC) (1.2%). For pain score, procedure time, and time to healing, lower values represented better outcomes; rankings for these endpoints were therefore interpreted in that direction. Enzymatic and autolytic debridement ranked more favorably for pain score, biological and ultrasound-assisted debridement (US) for procedure time, and biological and autolytic debridement for time to healing. Several comparisons had wide credible intervals, and certainty of evidence was limited by clinical heterogeneity, sparse networks, and methodological limitations.

**Conclusion:**

The comparative profile of debridement strategies varied by the outcome. Biological and enzymatic debridement ranked favorably for wound healing and complete debridement, whereas enzymatic and autolytic debridement were associated with lower pain scores. Ultrasound-assisted debridement may be more efficient in terms of procedure time. These findings should be interpreted cautiously because ranking probabilities do not establish clinically decisive superiority, especially when evidence is sparse or heterogeneous.

**Systematic review registration:**

https://www.crd.york.ac.uk/prospero/display_record.php?ID=CRD420261341535.

## 1 Introduction

Clinically, chronic lower-extremity wounds are commonly described as wounds that fail to heal after more than 1 month of standard care or show no clear signs of progression toward healing. Chronic ulcers mainly include venous leg ulcers, arterial or ischemic ulcers, mixed arterial–venous ulcers, and diabetic foot ulcers (1). Normal wound healing has four overlapping phases: hemostasis, inflammation, proliferation, and remodeling. It relies on the coordinated activity of repair cells, inflammatory cells, the extracellular matrix, and multiple growth factors and cytokines (2). By contrast, chronic lower-extremity wounds are characterized by persistent inflammation, impaired perfusion, and disrupted neovascularization, all of which delay normal tissue repair. Sustained inflammation plays a central role by inhibiting progression to the proliferative phase. In particular, excessive neutrophil infiltration contributes to this dysregulated inflammatory state; overactivation of neutrophils promotes the formation of neutrophil extracellular traps, which further delay wound repair (3, 4).

Chronic lower-extremity wounds are associated with high prevalence, complex etiology, prolonged hospitalization, frequent recurrence, and substantial risks of disability and mortality. They significantly reduce patients’ quality of life and impose a considerable clinical and economic burden on healthcare systems worldwide. In the United States, approximately 10.5 million Medicare beneficiaries are affected, with total costs exceeding $50 billion (5). In China, chronic lower-extremity wounds account for an estimated 30 million treatment visits annually. Moreover, population aging and shifts in disease patterns are contributing to a steady rise in incidence, further increasing the societal burden (6).

Advances in understanding the mechanisms underlying impaired wound healing have led to substantial progress in therapeutic approaches. Novel wound dressings with improved antimicrobial and breathable properties have largely replaced traditional materials, while the use of biologics and growth factors has enhanced both the rate and quality of healing. Current clinical strategies include surgical debridement (SURG), negative pressure wound therapy (NPWT), skin or flap grafting, growth factor application, mesenchymal stem cell therapy, and hyperbaric oxygen therapy (7). Among these, debridement remains the cornerstone of chronic wound management, forming the basis of most treatment protocols. It is essential for effective wound bed preparation, as necrotic tissue impairs healing and obstructs keratinocyte migration. The removal of devitalized tissue and local bacterial burden creates a more favorable environment for wound closure (8).

A range of debridement techniques is currently used in clinical practice, including surgical (sharp), mechanical (e.g., wet dressings, irrigation, or monofilament pads), enzymatic (e.g., collagenase and papain), and biological methods (e.g., maggot therapy). These approaches differ in mechanisms, indications, and clinical effectiveness (9). Evidence comparing their relative efficacy remains inconsistent, and the majority of randomized controlled trials have been limited to pairwise comparisons, hindering comprehensive evaluation (10). Network meta-analysis enables the simultaneous comparison of multiple interventions within a unified framework and allows estimation of their relative effectiveness through probabilistic ranking. Therefore, a network meta-analysis may provide a more comprehensive comparison of debridement strategies and help inform evidence-based wound care decisions.

## 2 Methods

### 2.1 Study design

This study was conducted in accordance with the Preferred Reporting Items for Systematic Reviews and Meta-Analyses extension statement for Network Meta-Analyses (PRISMA-NMA) guidelines. This study was previously registered with the International Registry for Prospective Systematic Reviews (PROSPERO registration number: CRD420261341535).

### 2.2 Search strategy

Two researchers independently searched PubMed, Web of Science, Embase, the Cochrane Library, the China National Knowledge Infrastructure (CNKI), Wanfang, and China Science and Technology Journal (VIP) databases. The search was conducted in titles, abstracts, and keywords and covered studies published from January 1990 to February 2026. Only articles published in English or Chinese were considered. The search strategy focused on four main concepts: chronic lower-extremity wounds, debridement strategies, wound management, and randomized controlled trials. Terms related to the study population included chronic wound, chronic ulcer, venous leg ulcer, arterial ulcer, ischemic ulcer, mixed arterial–venous ulcer, diabetic foot ulcer, lower-extremity wound, lower-extremity ulcer, and related conditions. Intervention-related terms included autolytic debridement, enzymatic debridement, biological debridement, mechanical debridement, ultrasound-assisted debridement, surgical or sharp debridement, and hydrosurgical debridement (HYDRO). Methodological terms included randomized controlled trial, randomized, and clinical trial. Medical Subject Headings and free-text terms were combined using Boolean operators to improve search sensitivity. In addition, the reference lists of the included studies were manually screened to identify other potentially relevant articles.

### 2.3 Inclusion and exclusion criteria

*Studies were included if they met all the following criteria:*

(1) The study design was a randomized controlled trial (RCT) or cluster randomized controlled trial;
(2) Participants had chronic lower-extremity wounds or ulcers, including venous leg ulcers, arterial or ischemic ulcers, mixed arterial–venous ulcers, diabetic foot ulcers, and other chronic lower-extremity wounds as defined by the original studies;
(3) The wound was characterized by necrotic tissue, slough, fibrin, or other non-viable tissue;
(4) The intervention compared different debridement approaches, including autolytic, enzymatic, biological, mechanical, ultrasound-assisted, surgical or sharp, and hydrosurgical debridement;
(5) At least one extractable outcome related to wound healing or debridement was reported, such as wound healing, complete debridement, pain score, procedure time, or time to healing.

*Studies were excluded if they met any of the following criteria:*

(1) Non-randomized designs, including cohort studies, case–control studies, case series, case reports, animal studies, systematic reviews, or conference abstracts;
(2) Acute traumatic wounds.
(3) Evaluation of non-debridement interventions only, including dressings alone, growth factors, negative pressure wound therapy, stem cell therapy, shock wave therapy, or other non-debridement treatments;
(4) Mixed wound populations from which data specific to eligible chronic ulcer types could not be extracted separately;
(5) Incomplete outcome data that could not be extracted or calculated, duplicate publications, or studies with overlapping datasets.

### 2.4 Literature screening and data extraction

Two reviewers independently screened the retrieved records according to the predefined eligibility criteria. Disagreements were resolved through discussion or consultation with a third reviewer. Data were extracted using a standardized form by one reviewer and checked independently by another reviewer. Extracted information included first author, publication year, country, study design, wound type, sample size, participant age and sex, intervention and comparator, debridement category, treatment duration or frequency, follow-up duration, and reported outcomes. The outcomes extracted for analysis included wound healing, complete debridement, procedure time, time to healing, and pain score.

### 2.5 Risk of bias assessments

The methodological quality of the included studies was independently assessed by two reviewers. The Cochrane RoB 2.0 was used to evaluate the risk of bias of the included randomized controlled trials. RoB 2.0 assesses five domains: bias arising from the randomization process, bias due to deviations from intended interventions, bias due to missing outcome data, bias in measurement of the outcome, and bias in selection of the reported result. Each domain was judged as “low risk of bias,” “some concerns,” or “high risk of bias.” Based on the assessments across all domains, an overall risk of bias judgment was assigned for each study. The assessment results were summarized and presented in graphical form to illustrate the distribution of bias risk across the included studies and domains.

### 2.6 Transitivity assessment

Before conducting the network meta-analysis, we assessed the transitivity assumption by comparing the distribution of clinically relevant effect modifiers across treatment comparisons and treatment nodes. The evaluated effect modifiers included wound etiology, baseline wound severity, wound size and duration, infection status, necrotic or slough burden, vascular status, participant age and sex distribution, intervention frequency and duration, follow-up duration, outcome definitions, co-interventions, and risk of bias. These factors are summarized in Supplementary Table 1 and assessed qualitatively because several variables were not consistently reported across studies.

### 2.7 Statistical analysis

Bayesian network meta-analysis was conducted using Markov chain Monte Carlo methods in R, mainly with the gemtc, rjags, coda, and BUGSnet packages. For dichotomous outcomes, a binomial likelihood with a log link was used, and the results were expressed as risk ratios (RRs) with 95% credible intervals (CrIs). For continuous outcomes, a normal likelihood with an identity link was used, and the results were expressed as mean differences (MDs) or standardized mean differences (SMDs), as appropriate. Random-effects consistency models were used as the primary models because clinical heterogeneity was expected across trials. Fixed-effect models were also fitted where appropriate for model fit comparison. Model fit was assessed using the deviance information criterion and residual deviance. Vague or non-informative priors were assigned to treatment effects, and a weakly informative prior was assigned to the between-study heterogeneity parameter. For the main gemtc analyses, four Markov chains were run with 5,000 adaptation iterations and 20,000 sampling iterations. For BUGSnet-based model fit diagnostics, models were run with 1,000 adaptation iterations, 10,000 burn-in iterations, and 50,000 sampling iterations. Adequacy of the chain length was evaluated using convergence diagnostics, including trace plots, density plots, autocorrelation plots, and potential scale reduction factor (PSRF)/R-hat convergence statistic (Rhat) values. Treatment rankings were interpreted according to the clinical direction of each outcome. For wound healing and complete debridement, higher values indicated greater benefit; therefore, higher SUCRA values represented more favorable rankings. For pain score, procedure time, and time to healing, lower values indicated greater benefit; therefore, lower SUCRA values represented more favorable rankings when SUCRA was calculated on the original outcome scale. To avoid misinterpretation, all ranking results for these outcomes were reported and discussed according to this predefined direction of clinical benefit rather than according to a uniform “higher-is-better” rule. Local inconsistency was assessed using node-splitting analyses where the evidence network allowed. Pairwise meta-analyses were performed using STATA version 16.0. For dichotomous outcomes, treatment effects were estimated as risk ratios (RRs) with 95% confidence intervals (CIs).

## 3 Results

### 3.1 Literature search results

**Figure 1:**
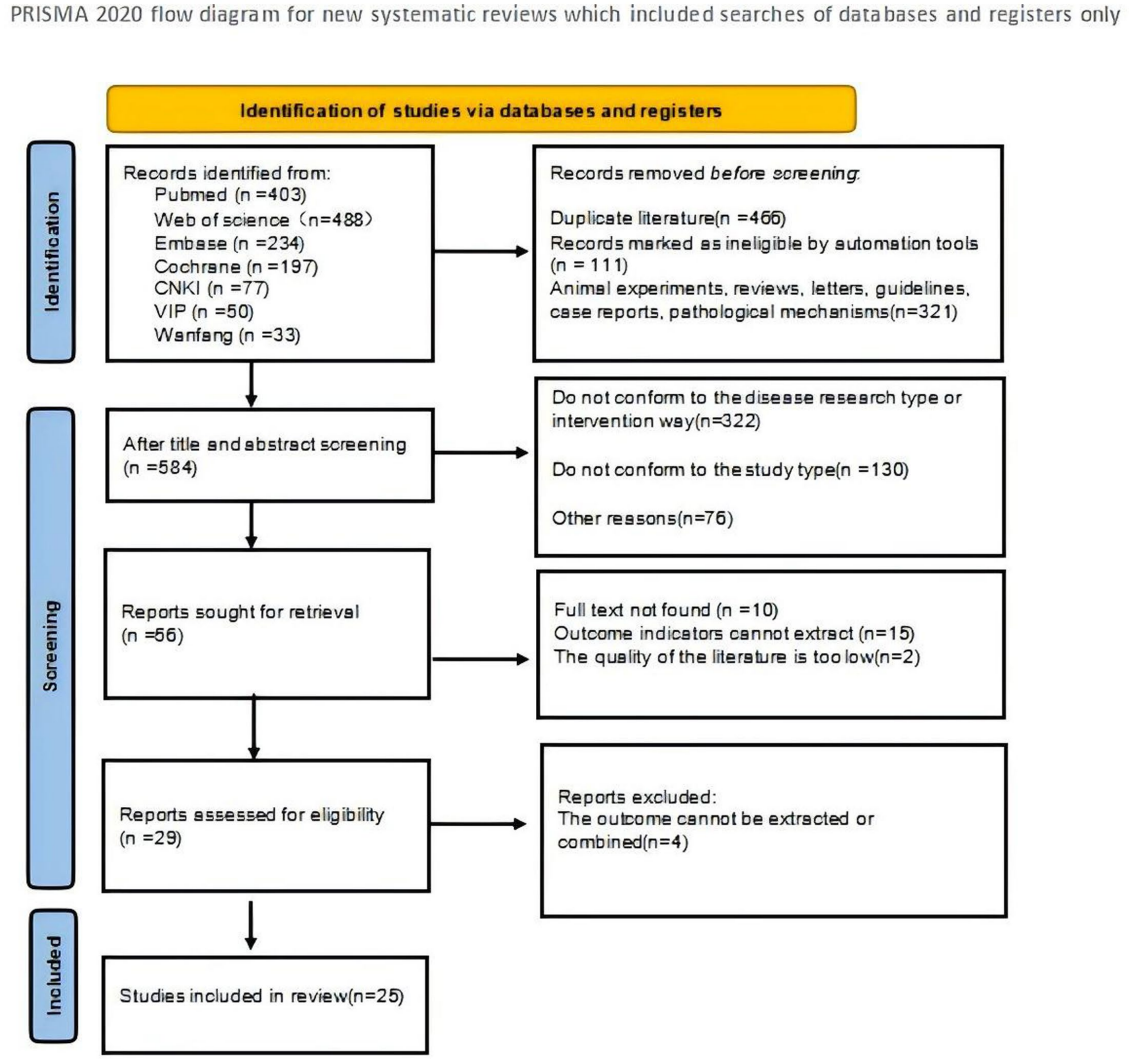
Literature screening process.

A total of 1,482 records were identified from PubMed (*n* = 403), Web of Science (*n* = 488), Embase (*n* = 234), the Cochrane Library (*n* = 197), CNKI (*n* = 77), VIP (*n* = 50), and Wanfang (*n* = 33). Before screening, 898 records were removed, including duplicate records (*n* = 466), records marked as ineligible by automation tools (*n* = 111), and clearly irrelevant records such as animal experiments, reviews, letters, guidelines, case reports, and pathological mechanism studies (*n* = 321). After title and abstract screening, 584 records remained. Of these, 528 records were excluded because they did not meet the predefined criteria, including studies with irrelevant disease types or interventions (*n* = 322), inappropriate study designs (*n* = 130), and other reasons (*n* = 76). Subsequently, 56 reports were sought for retrieval. After excluding reports with unavailable full text (*n* = 10), non-extractable outcome data (*n* = 15), or low methodological quality (*n* = 2), 29 reports were assessed for eligibility. Four reports were further excluded because the outcomes could not be extracted or combined. Finally, 25 randomized controlled trials were included in the network meta-analysis. The study selection process is shown in Figure 1.

### 3.2 Basic characteristics of the included studies

A total of 25 randomized controlled trials involving 1,756 patients with chronic lower-extremity wounds were included in this study. The characteristics of the included studies, baseline information of participants, and details of the interventions and control treatments are presented in Table 1.

**Table 1:** Clinical and demographic characteristics of the included studies in NMA.

Author	Year	Country	Study type	Causes of chronic lower-extremity wounds	Intervention		Sample size (*n*)		Age (years)		Sex (male/female)	
					Experimental	Control	Experimental	Control	Experimental	Control	Experimental	Control
Saranholi et al. (26)	2025	Brazil	RCT	Venous leg ulcers	Mechanical	Enzymatic	28	28	68.6	70.7	NA	NA
Shoham et al. (27)	2024	Multicenter	RCT	Venous leg ulcers	Enzymatic	Autolytic	46	43	65.5	61.4	26/20	27/16
	2024	Multicenter	RCT	Venous leg ulcers	Enzymatic	Standard care	46	30	65.5	65.5	26/20	11/19
Cangel et al. (28)	2022	Turkey	RCT	Arterial ischemic ulcers	Biological	Standard care	39	33	NA	NA	NA	NA
Shoham et al. (29)	2021	Multicenter	RCT	venous leg ulcers (VLU); diabetic foot ulcers (DFU); post-trauma ulcers	Enzymatic	Autolytic	49	24	65.8	68.1	NA	NA
Alvarez et al. (30)	2019	USA	RCT	Venous leg ulcers	Ultrasound-assisted debridement	Surgical debridement	36	40	64	60	NA	NA
Colenci et al. (31)	2019	Brazil	RCT	Venous leg ulcers	Autolytic	Enzymatic	37	36	66	67.5	NA	NA
Michailidis et al. (32)	2018	Australia	RCT	Diabetic foot ulcers	Ultrasound-assisted debridement	Surgical debridement	7	7	NA	NA	NA	NA
deAraujo et al. (33)	2016	Brazil	RCT	Venous leg ulcers	Autolytic	Standard care	21	23	62	62	NA	NA
	2016	Brazil	RCT	Venous leg ulcers	Enzymatic	Standard care	19	23	62	62	NA	NA
Liu et al. (34)	2015	USA	RCT	Chronic lower-extremity wounds	Hydrosurgical	Surgical debridement	21	19	52.2	57.1	16/5	10/9
Rodrigues et al. (35)	2015	Brazil	RCT	Venous leg ulcers	Enzymatic	Autolytic	16	12	61.9	61.9	NA	NA
Davies et al. (36)	2014	UK	RCT	Venous leg ulcers	Biological	Standard care	20	20	78.1	75.6	NA	NA
Humbert et al. (37)	2014	France	RCT	Venous leg ulcers	Autolytic	Standard care	34	41	74.8	73.7	NA	NA
Mudge et al. (38)	2014	UK	RCT	Venous/mixed leg ulcers	Biological	Autolytic	46	42	NA	NA	NA	NA
Tallis et al. (39)	2013	USA	Multicenter RCT	Diabetic foot ulcers	Enzymatic	Mechanical	24	24	61	61	16/8	16/8
Opletalova et al. (40)	2012	France	Multicenter RCT	Venous leg ulcers	Biological	Surgical debridement	51	54	72.8	73.9	22/29	23/31
Herberger et al. (41)	2011	Germany	RCT	Chronic vascular leg ulcers	Ultrasound-assisted debridement	Surgical debridement	34	33	74.5	70.5	NA	NA
Dumville et al. (42)	2009	UK	RCT	Venous/mixed leg ulcers	Biological	Autolytic	180	87	73.8	74.3	NA	NA
Caputo et al. (43)	2008	USA	RCT	Diabetic foot ulcers; venous ulcers	Hydrosurgical	Surgical debridement	22	19	68.5	67.6	NA	NA
König et al. (44)	2005	Germany	RCT	Venous leg ulcers	Autolytic	Enzymatic	15	27	71.7	71.7	NA	NA
Wayman et al. (45)	2000	UK	RCT	Venous leg ulcers	Biological	Autolytic	6	6	58	54	NA	NA
Westerhof et al. (46)	1990	Netherlands	RCT	Venous leg ulcers	Enzymatic	Autolytic	16	15	NA	NA	NA	NA
Lazaro-Martinez et al. (47)	2020	Spain	RCT	Diabetic foot ulcer	Ultrasound-assisted debridement	Standard care	27	24	64.1 ± 12.4	58 ± 5.4	3/24	24/0
Rastogi et al. (48)	2019	India	RCT	Diabetic foot ulcer	Ultrasound-assisted debridement	Standard care	34	26	52.5 ± 7.2	51.2 ± 7.3	NA	NA
Cao et al. (49)	2010	China	RCT	Diabetic foot ulcer	Ultrasound-assisted debridement	Standard care	12	12	58.1 ± 8.3	57.9 ± 8.2	NA	NA
Zu (50)	2019	China	RCT	Diabetic foot ulcer	Ultrasound-assisted debridement	Standard care	120	120	63.9 ± 5.5	62.8 ± 5.3	68/52	66/54

### 3.3 The quality assessment of the included studies

**Figure 2:**
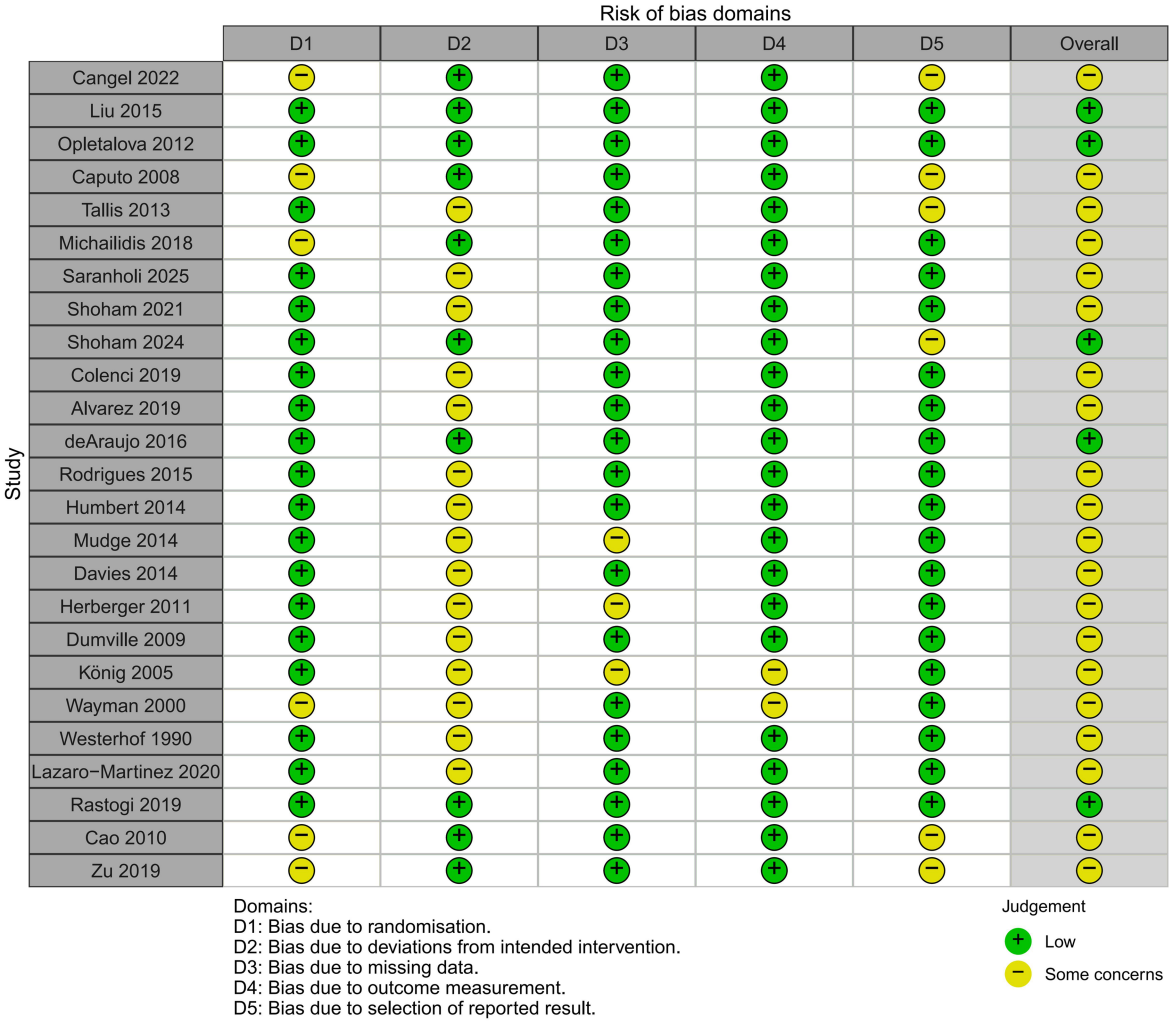
Risk of bias in randomized controlled trials.

A total of 25 RCTs were assessed using the Cochrane Risk of Bias 2.0 tool. Overall, methodological limitations were common across the included studies. Five studies were judged as having a low overall risk of bias, 20 studies were judged as having some concerns, and no study was judged as having a high overall risk of bias. At the domain level, 19 studies were rated as low risk, while six were rated as having some concerns for bias arising from the randomization process (D1), mainly because random sequence generation or allocation concealment was insufficiently described in some trials. For deviations from intended interventions (D2), 10 studies were rated as low risk, while 15 were rated as having some concerns, largely because blinding was difficult or incompletely reported in trials of debridement strategies. For missing outcome data (D3), 22 studies were rated as low risk, while three were rated as having some concerns. For measurement of the outcome (D4), 23 studies were rated as low risk, while two were rated as having some concerns. For selection of the reported result (D5), 19 studies were rated as low risk, while six were rated as having some concerns, mainly because trial protocols or prespecified statistical analysis plans were not consistently available. No study was rated as high risk in any overall judgment. The domain-level distribution of RoB 2.0 judgments is presented in Figure 2. A formal sensitivity analysis excluding studies at high risk of bias was not performed because no included trial was judged as having an overall high risk of bias. We also did not exclude all studies rated as having some concerns because this would have removed a large proportion of the available evidence, disconnected several treatment nodes, and made some outcome-specific networks unstable or non-estimable. Therefore, the risk of bias was incorporated into the Grading of Recommendations Assessment, Development, and Evaluation (GRADE) certainty assessment and considered when interpreting the robustness of the network estimates and treatment rankings.

## 4 Network meta-analysis

### 4.1 Intervention classification and node merging

To improve interpretability and preserve network connectivity, interventions were grouped into eight nodes according to their dominant mechanism and clinical implementation: autolytic debridement (AUTO), enzymatic debridement (ENZ), biological debridement (BIO), mechanical debridement (MECH), ultrasound-assisted debridement (US), sharp/surgical debridement (SURG), hydrosurgical debridement (HYDRO), and standard care (SC). AUTO included moisture-retentive approaches that promote endogenous breakdown of devitalized tissue. ENZ included exogenous proteolytic agents, such as collagenase-, papain-, or bromelain-based products. BIO referred to larval or maggot debridement therapy. MECH included non-ultrasound mechanical methods that remove non-viable tissue through physical force. US referred to ultrasound-assisted debridement techniques. SURG included sharp or conventional surgical debridement. HYDRO referred to hydrosurgical or waterjet-based debridement. SC included standard wound care, usual care, standard therapy, or conventional non-debridement comparator care as defined in the original trials. Since these comparator arms varied in dressings, compression therapy, offloading, infection control, and other background care, SC should be interpreted as a pragmatic comparator rather than a uniform intervention.

### 4.2 Wound healing

**Figure 3:**
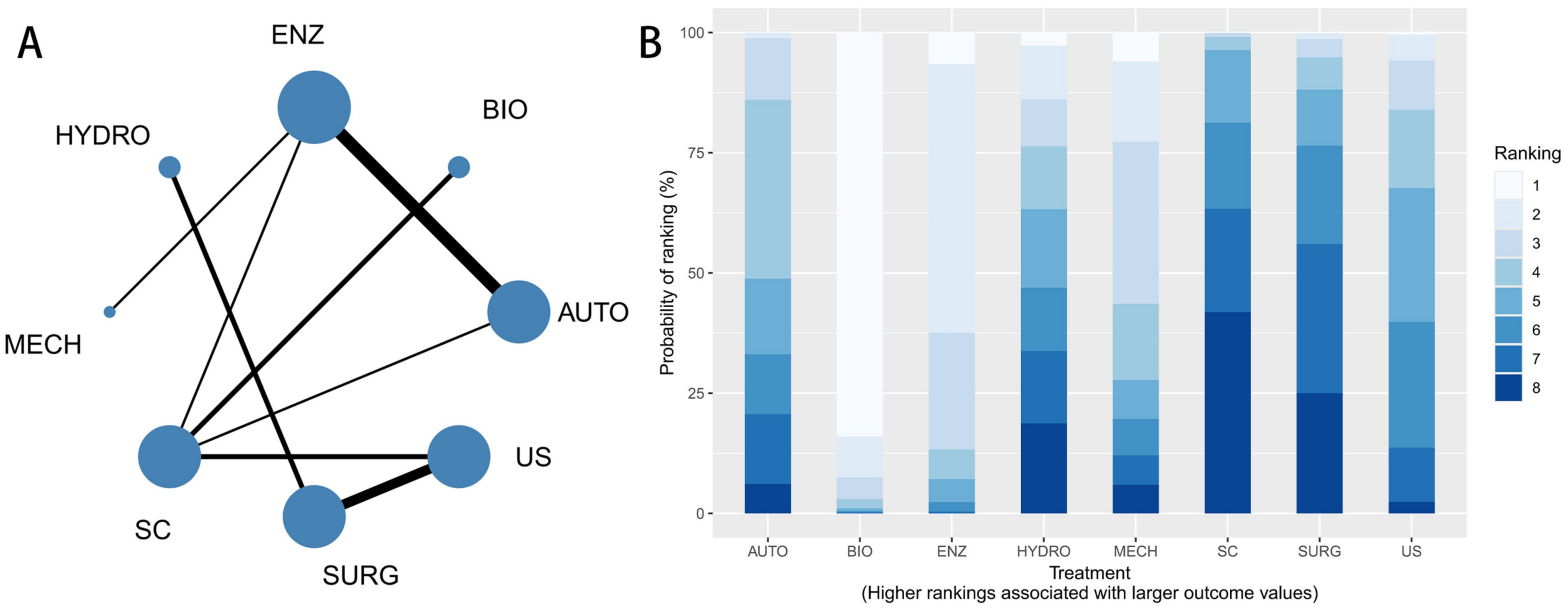
Network meta-analysis for wound healing. **(A)** Network evidence diagram for wound healing. Node size represents the amount of evidence for each intervention, and line thickness represents the number of direct comparisons. **(B)** Cumulative ranking probability plot for wound healing across AUTO, ENZ, BIO, MECH, US, SURG, HYDRO, and SC.

The network structure of the included interventions is shown in Figure 3A. A total of eight interventions were included in the network meta-analysis: AUTO, ENZ, BIO, MECH, US, SURG, HYDRO, and SC. In the network plot, each node represents a treatment, and the size of the node reflects the number of studies evaluating that intervention. The lines connecting nodes indicate direct comparisons between treatments, while thicker lines represent a larger number of head-to-head trials. The ranking probabilities of the interventions are presented in Figure 3B. The stacked bar chart illustrates the probability of each treatment being ranked from best to worst. Treatments with higher probabilities of occupying the top ranks are considered more likely to provide favorable outcomes. In addition, node-splitting analysis indicated no significant differences between direct and indirect comparisons (*p* > 0.05), supporting the assumption of consistency within the network (Supplementary Figure 7).

#### 4.2.1 Network meta-analysis pairwise comparisons

The league table for wound healing is shown in Supplementary Figure 2. Effect estimates were expressed as RRs with 95% CrIs, and an RR greater than 1 indicated a higher probability of wound healing. Compared with standard care, biological debridement showed the most favorable effect (RR = 4.56, 95% CrI 2.02–9.27), followed by enzymatic debridement (RR = 2.39, 95% CrI 1.16–4.58). Among active interventions, biological debridement was more favorable than autolytic debridement (RR = 3.39, 95% CrI 1.06–8.28), sharp/surgical debridement (RR = 4.63, 95% CrI 1.23–12.31), and ultrasound-assisted debridement (RR = 3.74, 95% CrI 1.10–9.35). Enzymatic debridement was also more favorable than autolytic debridement (RR = 1.60, 95% CrI 1.06–2.41). Other comparisons showed wide CrIs crossing the null value, indicating uncertainty.

#### 4.2.2 Efficacy ranking

The SUCRA ranking results were as follows: BIO (99.5%) > ENZ (83.1%) > MECH (65.9%) > AUTO (44.1%) > US (42.8%) > HYDRO (34.6%) > SURG (20.7%) > SC (9.2%). A higher SUCRA value indicates a greater probability that the intervention is more effective. These results suggested that biological debridement had the highest probability of being the most effective intervention for wound healing, followed by enzymatic and mechanical debridement.

### 4.3 Complete debridement

#### 4.3.1 Network evidence graph

**Figure 4:**
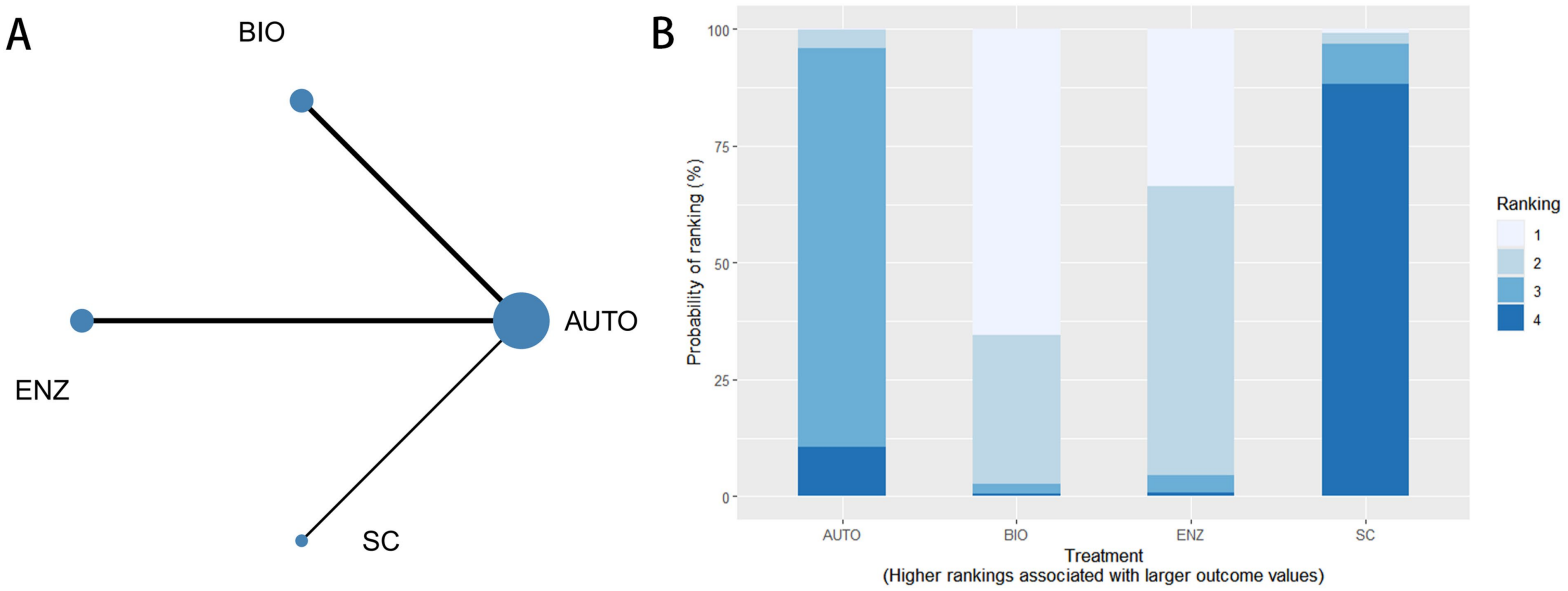
Network meta-analysis for complete debridement. **(A)** Network evidence diagram for complete debridement among AUTO, BIO, ENZ, and SC. **(B)** Cumulative ranking probability plot for complete debridement.

Four interventions (AUTO, BIO, ENZ, and SC) were included in the network meta-analysis for complete debridement. The evidence network is presented in Figure 4A, where each node represents an intervention and the connecting lines indicate direct comparisons between treatments. The ranking probabilities of the interventions are presented in Figure 4B.

#### 4.3.2 NMA

The league table for complete debridement is shown in Supplementary Figure 3. Effect estimates were expressed as RRs with 95% CrIs, and an RR greater than 1 indicated a higher probability of complete debridement. Compared with standard care, biological debridement showed a more favorable effect (RR = 6.26, 95% CrI 1.13–21.71). Enzymatic debridement also showed a favorable numerical effect compared with standard care, but the estimate was imprecise (RR = 5.16, 95% CrI 0.99–17.43). Among active interventions, biological debridement was more favorable than autolytic debridement (RR = 2.48, 95% CrI 1.07–5.04). Other comparisons had wide CrIs and should be interpreted cautiously.

#### 4.3.3 Efficacy ranking

Because lower pain scores indicate better tolerability, interventions with lower SUCRA values were interpreted as more favorable for this outcome. The ranking order was ENZ (18.2%), AUTO (19.5%), SC (30.3%), BIO (60.5%), SURG (73.6%), and US (97.9%).

### 4.4 Pain score

#### 4.4.1 Network evidence graph

**Figure 5:**
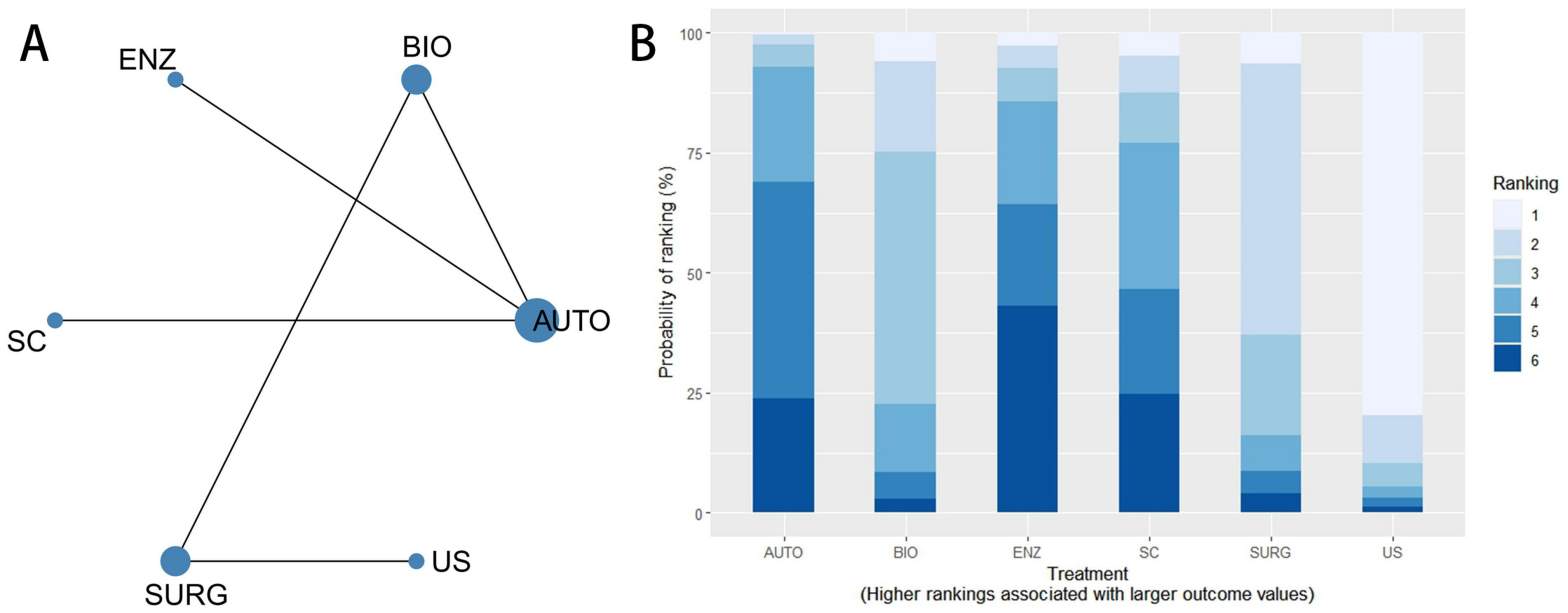
Network meta-analysis for pain score. **(A)** Network evidence diagram for pain score among AUTO, BIO, ENZ, SC, SURG, and US. **(B)** Cumulative ranking probability plot for pain score; rankings were interpreted according to the clinical direction that lower pain scores indicate better tolerability.

Six interventions (AUTO, BIO, ENZ, SC, SURG, and US) were included in the network meta-analysis for pain score. The evidence network is presented in Figure 5A, where nodes represent interventions and connecting lines indicate direct comparisons between treatments. The ranking probabilities of the interventions are presented in Figure 5B.

#### 4.4.2 NMA

The league table for pain score is shown in Supplementary Figure 4. Effect estimates were expressed as MDs with 95% CrIs. Since lower pain scores indicate better tolerability, negative MDs favored the intervention listed in the comparison. Enzymatic and autolytic debridement showed numerically lower pain scores than several other interventions. For example, compared with ultrasound-assisted debridement, enzymatic debridement (MD = −2.64, 95% CrI − 6.05 to 0.79) and autolytic debridement (MD = −2.54, 95% CrI − 5.53 to 0.45) showed lower pain scores. However, all pairwise comparisons had 95% CrIs crossing 0, indicating that no conclusive difference was observed for the pain score.

#### 4.4.3 Efficacy ranking

Since lower pain scores indicate better tolerability, lower SUCRA values represented more favorable rankings. The ranking results were ENZ (18.2%) < AUTO (19.5%) < SC (30.3%) < BIO (60.5%) < SURG (73.6%) < US (97.9%).

### 4.5 Procedure time

#### 4.5.1 Network evidence graph

**Figure 6:**
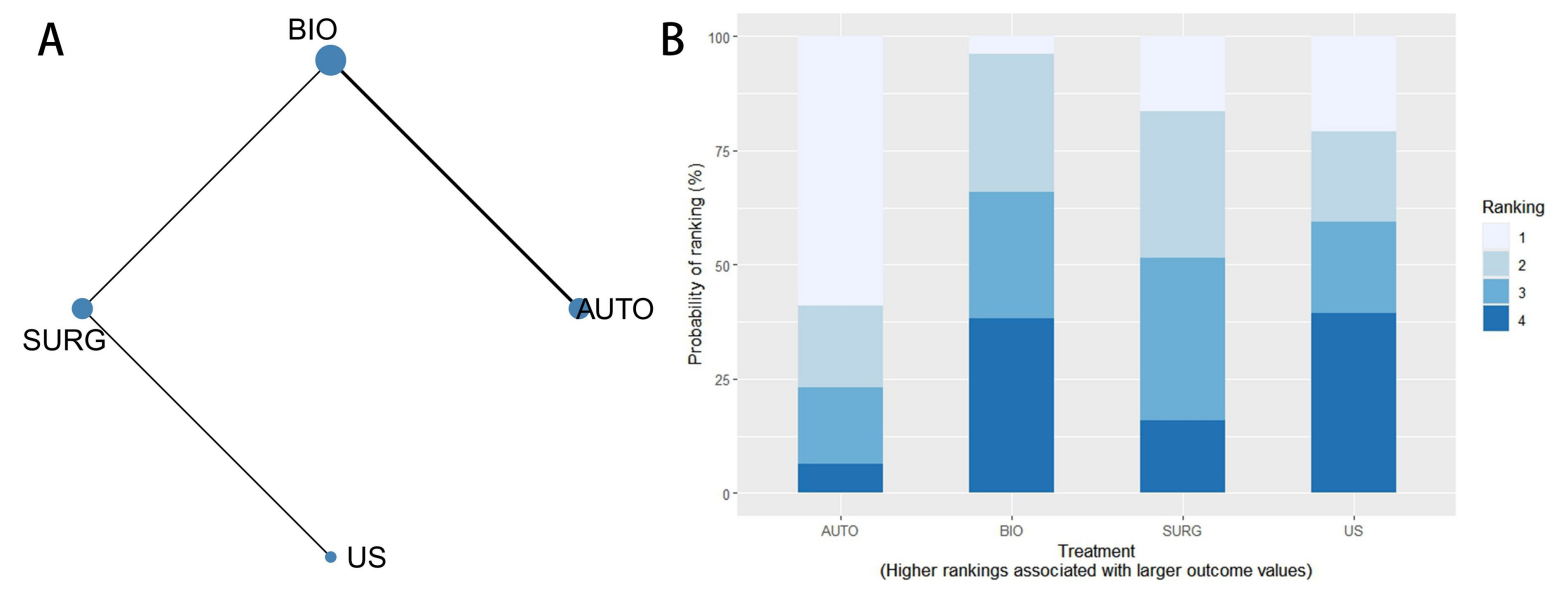
Network meta-analysis for procedure time. **(A)** Network evidence diagram for procedure time among AUTO, BIO, SURG, and US. **(B)** Cumulative ranking probability plot for procedure time; rankings were interpreted according to the clinical direction that shorter procedure time indicates better efficiency.

Four interventions (AUTO, BIO, SURG, and US) were included in the network meta-analysis for procedure time. The evidence network is shown in Figure 6A, where nodes represent interventions and connecting lines indicate direct comparisons between treatments. The ranking probabilities of the interventions are presented in Figure 6B.

#### 4.5.2 NMA

The league table for procedure time is shown in Supplementary Figure 5. Effect estimates were expressed as MDs with 95% CrIs. Since shorter procedure time indicates better efficiency, negative MDs favored the intervention listed in the comparison. Biological debridement showed a numerically shorter procedure time than autolytic debridement (MD = −9.98, 95% CrI − 29.26 to 8.42) and sharp/surgical debridement (MD = −2.88, 95% CrI − 28.77 to 22.82). Compared with ultrasound-assisted debridement, biological debridement also showed a small numerical advantage (MD = −0.80, 95% CrI − 37.82 to 34.77). However, all CrIs crossed 0, suggesting substantial uncertainty and no conclusive pairwise difference.

#### 4.5.3 Efficacy ranking

Because shorter procedure time indicates better efficiency, lower SUCRA values represented more favorable rankings. The ranking results were BIO (18.0%) < US (21.9%) < SURG (79.4%) < AUTO (80.7%).

### 4.6 Time to healing

#### 4.6.1 Network evidence graph

**Figure 7:**
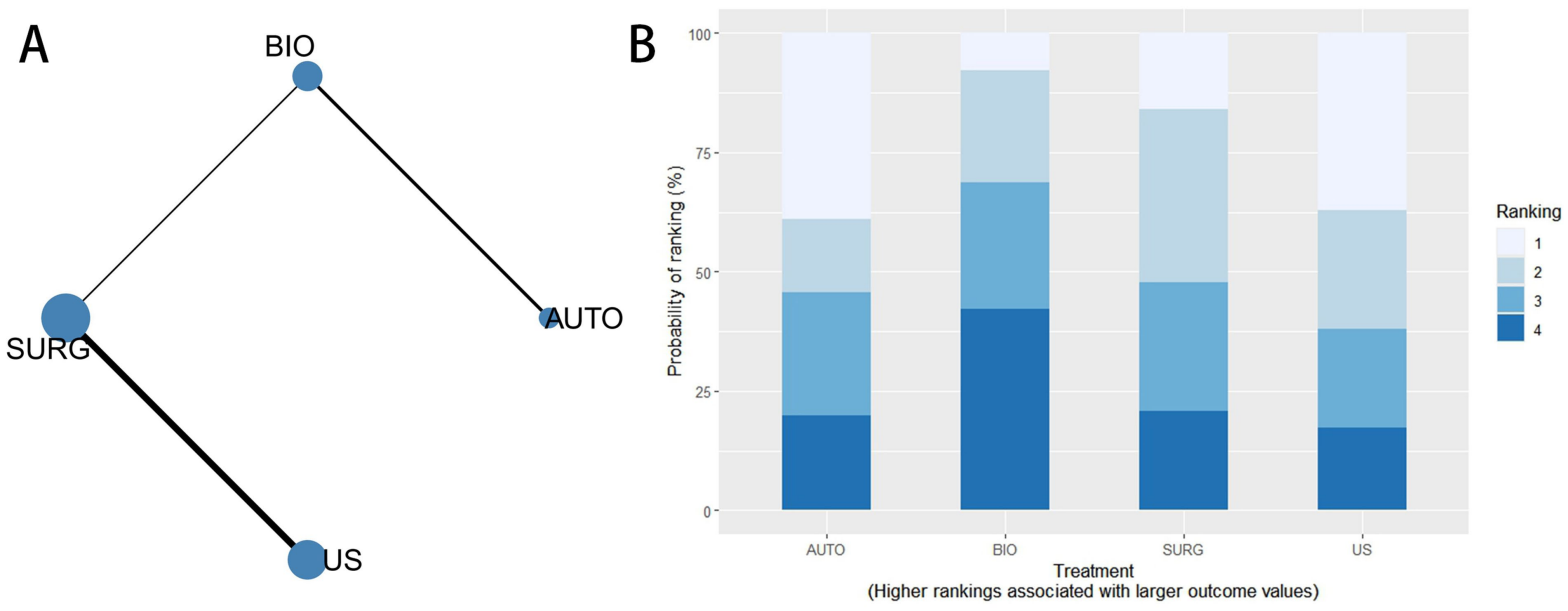
Network meta-analysis for time to healing. **(A)** Network evidence diagram for time to healing among AUTO, BIO, SURG, and US. **(B)** Cumulative ranking probability plot for time to healing; rankings were interpreted according to the clinical direction that shorter time to healing indicates a better outcome.

Four interventions (AUTO, BIO, SURG, and US) were included in the network meta-analysis for time to healing. The evidence network is shown in Figure **7**A, where nodes represent different interventions and connecting lines indicate direct comparisons between treatments. Consistency assessment indicated that the consistency model had a lower deviance information criterion (DIC) value than the inconsistency model, suggesting good agreement between direct and indirect evidence. The ranking probabilities of the interventions are presented in Figure **7**B.

#### 4.6.2 NMA

The league table for time to healing is shown in Supplementary Figure 6. Effect estimates were expressed as MDs with 95% CrIs. Since a shorter time to healing indicates a better outcome, negative MDs favored the intervention listed in the comparison. Biological debridement showed a numerically shorter time to healing than ultrasound-assisted debridement (MD = −13.59, 95% CrI − 88.03 to 56.32), autolytic debridement (MD = −10.69, 95% CrI − 56.46 to 34.84), and sharp/surgical debridement (MD = −8.39, 95% CrI − 72.81 to 55.39). However, all 95% CrIs were wide and crossed 0, indicating that the evidence for this outcome was sparse and uncertain.

#### 4.6.3 Efficacy ranking

Since a shorter time to healing indicates a better outcome, lower SUCRA values represented more favorable rankings. The ranking results were BIO (10.0%) < AUTO (33.3%) < SURG (66.7%) < US (91.0%).

### 4.7 Forest plots of the network meta-analysis for wound-related outcomes

**Figure 8:**
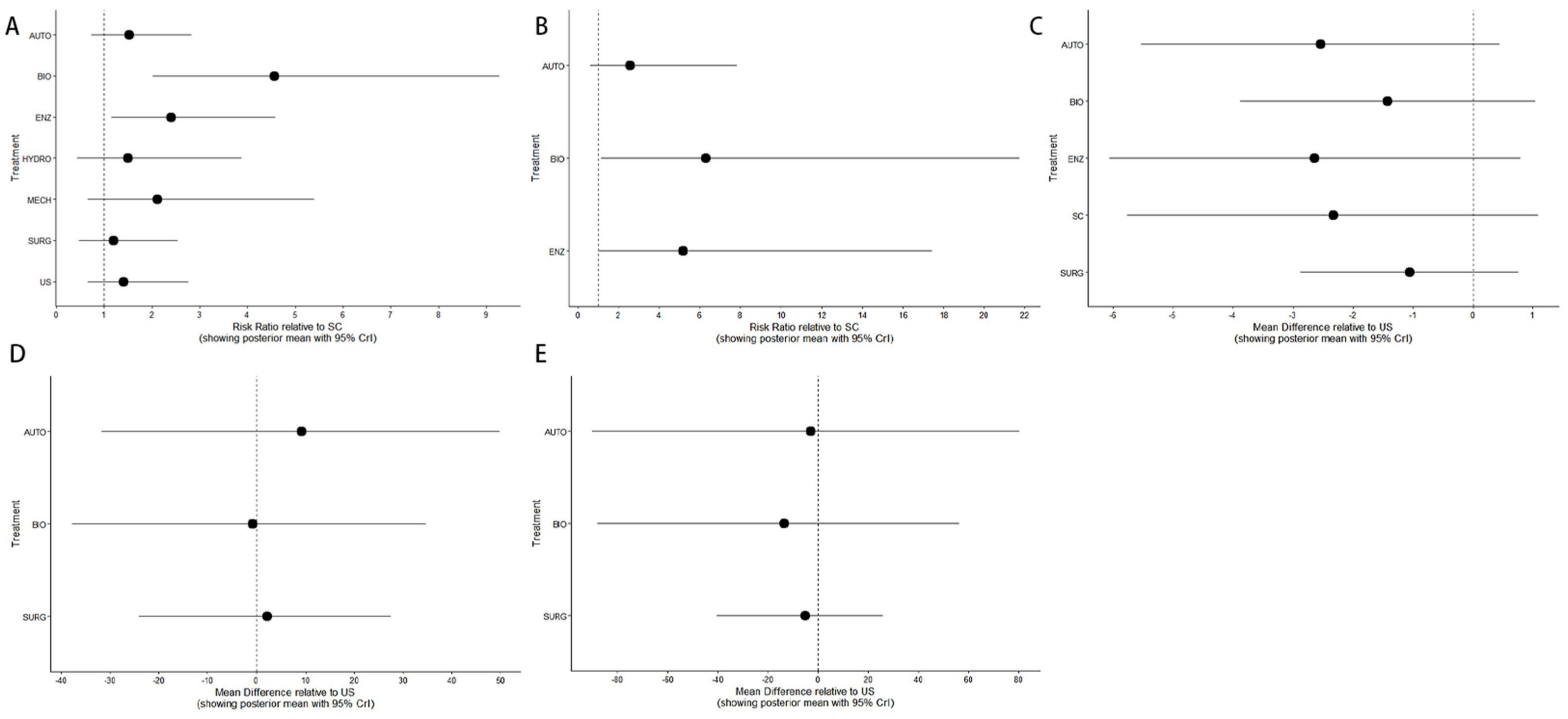
Forest plots of the network meta-analysis comparing different interventions. **(A)** Wound healing and **(B)** complete debridement are presented as risk ratios relative to SC. **(C)** Pain score, **(D)** procedure time, and **(E)** time to healing are presented as mean differences relative to US. Points represent posterior mean estimates, and horizontal lines indicate 95% credible intervals.

The forest plots comparing treatment effects across the included interventions are presented in Figure 8. For wound healing and complete debridement (Figures 8A–B), treatment effects were estimated as risk ratios relative to SC. For pain score, procedure time, and time to healing (Figures 8C–E), mean differences were calculated relative to US. Overall, the effect estimates varied across interventions and outcomes. Some debridement strategies demonstrated more favorable effects than the reference treatments, whereas others showed comparable or less pronounced effects. These findings indicate that the relative effectiveness of debridement strategies differs across clinically relevant outcomes.

### 4.8 Consistency test

**Figure 9:**
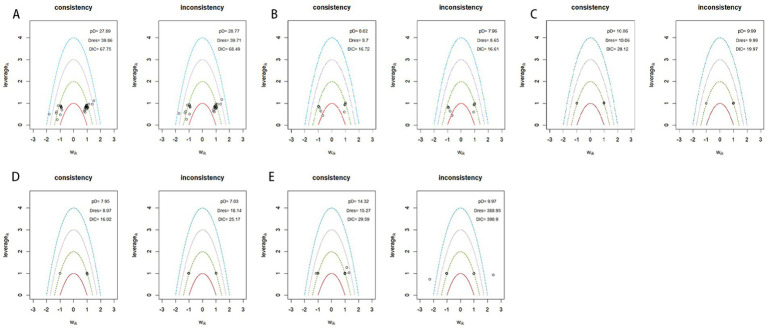
Consistency assessment comparing the consistency and inconsistency models for five outcomes: **(A)** Wound healing, **(B)** complete debridement, **(C)** pain score, **(D)** procedure time, and **(E)** time to healing.

Consistency between direct and indirect evidence was evaluated by comparing the consistency and inconsistency models. As shown in Figure 9, the consistency model showed lower DIC values for wound healing, procedure time, and time to healing. The DIC values were 67.75 vs. 68.48 for wound healing, 16.02 vs. 25.17 for procedure time, and 29.59 vs. 396.90 for time to healing. For pain scores, the inconsistency model showed a slightly lower DIC than the consistency model (19.87 vs. 20.12), but the difference was small and unlikely to indicate a meaningful improvement in model fit. For complete debridement, the inconsistency model also showed a lower DIC than the consistency model (14.61 vs. 18.32), suggesting that this outcome should be interpreted with caution. Overall, the residual deviance values were generally comparable between the two models for most outcomes, except for time to healing, where the inconsistency model showed a much poorer fit. In addition, node-splitting analyses showed no statistically significant inconsistency between direct and indirect comparisons where data were available. Therefore, the consistency assumption was considered broadly acceptable, although the results for complete debridement and pain score were interpreted cautiously because of sparse evidence and potential inconsistency.

### 4.9 SUCRA ranking probabilities from the network meta-analysis

**Figure 10:**
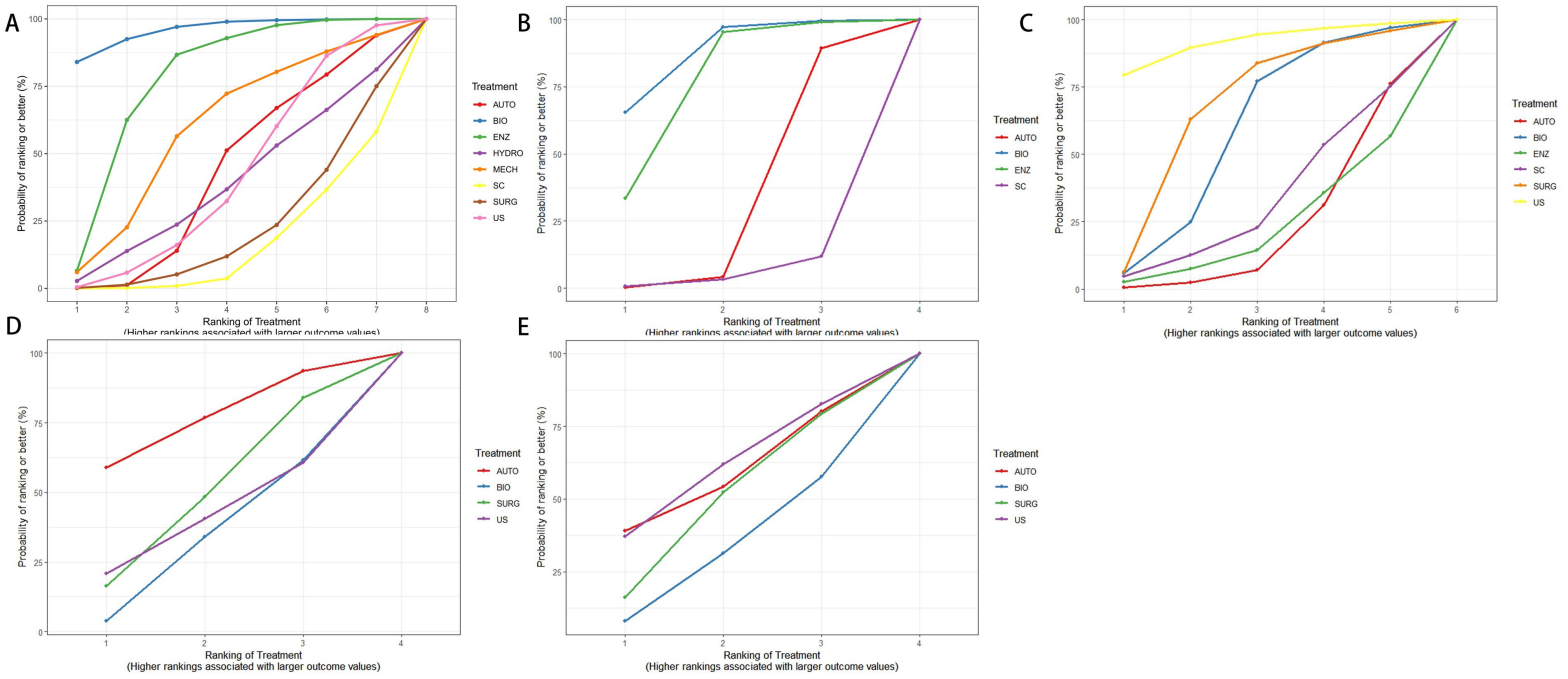
SUCRA ranking probabilities: **(A)** Wound healing; **(B)** complete debridement; **(C)** pain score; **(D)** procedure time; **(E)** time to healing.

SUCRA curves present the ranking probabilities of each intervention across outcomes. For wound healing and complete debridement, higher values indicated more favorable outcomes. For pain score, procedure time, and time to healing, lower values indicated more favorable outcomes; therefore, rankings for these outcomes were interpreted according to the direction of clinical benefit (Figure 10).

### 4.10 Transitivity assessment

The distribution of potential effect modifiers across treatment comparisons is summarized in Supplementary Table 1. The included studies mainly involved venous leg ulcers, diabetic foot ulcers, arterial or ischemic ulcers, and mixed arterial–venous ulcers. After exclusion of burn wounds, the clinical scope of the network was restricted to chronic lower-extremity wounds; however, important clinical heterogeneity remained across wound etiologies. Overall, the transitivity assumption was considered clinically plausible but only partially supported. Comparability was relatively better among studies of venous leg ulcers because several trials used similar vascular eligibility criteria, compression-based background care, and exclusion of severe arterial insufficiency or severe infection. In contrast, greater transitivity concerns were identified for diabetic foot ulcers and ischemic ulcers because these wounds may differ in neuropathy, perfusion status, infection burden, offloading requirements, revascularization status, and background standard care. Additional differences were observed in baseline wound size and duration, necrotic or slough burden, treatment frequency, co-interventions, follow-up duration, and outcome definitions. Since the evidence network became sparse when stratified by wound etiology, a fully stratified network meta-analysis by wound type was not considered statistically reliable. Therefore, conventional pairwise subgroup analyses by wound etiology were performed as exploratory analyses, and the overall network rankings were interpreted cautiously as ranking-based estimates rather than as definitive evidence of superiority across all wound types.

### 4.11 Node analysis and heterogeneity test

Node-splitting analysis and heterogeneity assessments were performed where the evidence network allowed. As shown in Supplementary Figure 7, node-splitting analysis for wound healing showed no statistically significant disagreement between direct and indirect evidence, with corresponding *p*-values greater than 0.05. Heterogeneity assessments are presented in Supplementary Figures 8, 9. Overall, the majority of direct comparisons showed low to moderate heterogeneity, although the interpretation of these findings should account for sparse evidence and clinical heterogeneity across wound types and intervention protocols.

### 4.12 Summary of findings and quality of evidence (GRADE)

The certainty of evidence for the main outcomes was assessed using the GRADE approach. For wound healing, 18 randomized controlled trials including 1,128 participants were available, and the overall certainty of evidence was moderate, downgraded due to imprecision. For complete debridement, five studies involving 335 participants provided low-certainty evidence, downgraded due to imprecision and potential inconsistency. Evidence for pain score was low certainty due to risk of bias and inconsistency across studies. For procedure time, the certainty of evidence was moderate, downgraded due to indirectness. Evidence for time to healing was rated as low certainty due to inconsistency and imprecision across studies (Supplementary Table 3).

### 4.13 Subgroup analyses based on conventional pairwise meta-analysis

To further explore the robustness of the network meta-analysis results, conventional pairwise meta-analyses were performed, and subgroup analyses were conducted according to wound etiology and type of debridement strategy (Supplementary Figure 10). When stratified by debridement strategy, biological debridement showed a significantly higher likelihood of wound healing compared with standard care (RR = 4.51, 95% CI 1.62–12.56), and enzymatic debridement also demonstrated a favorable effect compared with standard care (RR = 2.42, 95% CI 1.23–4.75). In contrast, hydrosurgical debridement vs. surgical debridement (RR = 1.10, 95% CI 0.67–1.79), ultrasound-assisted debridement vs. surgical debridement (RR = 1.23, 95% CI 0.88–1.71), ultrasound-assisted debridement vs. standard care (RR = 1.33, 95% CI 0.86–2.07), and autolytic debridement vs. standard care (RR = 1.04, 95% CI 0.70–1.54) did not show statistically significant differences. When stratified by wound etiology, the pooled results suggested that debridement strategies significantly improved wound healing in venous leg ulcers (RR = 1.53, 95% CI 1.08–2.16). A significant effect was also observed in arterial ischemic ulcers (RR = 7.62, 95% CI 3.03–19.17), although this estimate was based on a single study and should therefore be interpreted cautiously. No statistically significant differences were observed in diabetic foot ulcers or mixed leg ulcers. Overall, the random-effects model showed that debridement interventions were associated with a significantly higher likelihood of wound healing compared with control treatments (RR = 1.53, 95% CI 1.13–2.07), with substantial heterogeneity among studies (*I*^2^ = 72.3%). These findings were generally consistent with the network meta-analysis, supporting the potential benefits of debridement strategies, particularly biological and enzymatic approaches, in promoting wound healing.

## 5 Discussion

There is currently no universally accepted definition of a chronic wound. In clinical practice, it is generally described as a wound that fails to progress through the normal healing process because of various internal and external factors, remains in a prolonged inflammatory state, and does not heal despite conventional treatment. Common chronic lower-extremity wounds include diabetic foot ulcers, venous leg ulcers, arterial or ischemic ulcers, and mixed arterial–venous ulcers. Diabetic foot ulcers represent one of the most serious complications of diabetes and affect approximately 18.6 million individuals worldwide. Without timely intervention, they may progress to infection, gangrene, or amputation. Venous ulcers account for the majority of chronic lower-limb wounds, with a prevalence of approximately 1–2%. They are more common in older adults and women and are typically characterized by large but relatively shallow lesions. Although arterial ulcers are less common, they are often associated with inadequate arterial perfusion and are more difficult to manage. With the aging of the population, the incidence of chronic lower-extremity wounds continues to increase, placing an increasing burden on healthcare systems.

Debridement is the most fundamental and critical step in the management of chronic lower-extremity wounds. Its primary functions are to remove necrotic tissue, reduce bacterial burden, and prepare a favorable wound bed for healing. A wide range of debridement methods is currently available in clinical practice, including surgical and mechanical approaches; physical techniques such as ultrasound-assisted debridement and waterjet debridement; and autolytic, enzymatic, and biological methods. Given the diversity of these approaches and the uncertainty regarding their comparative effectiveness, this study used a Bayesian network meta-analysis to systematically evaluate and compare the relative efficacy of different debridement strategies.

This study included 25 randomized controlled trials involving 1,756 patients and compared multiple debridement strategies across several clinically relevant outcomes. The network meta-analysis suggests that the relative effectiveness of debridement strategies differed by outcome. For wound healing and complete debridement, biological debridement and enzymatic debridement ranked more favorably than several other interventions. For pain score, autolytic debridement and enzymatic debridement appeared to be associated with lower pain levels. For procedure time, ultrasound-assisted debridement and biological debridement ranked more favorably, whereas for time to healing, biological debridement and autolytic debridement ranked favorably. According to the GRADE assessment, the certainty of evidence varied across outcomes and was limited for several comparisons, particularly complete debridement, pain score, and time to healing. Therefore, although the ranking results suggest potential advantages for several strategies, these findings should be interpreted cautiously in light of heterogeneity in wound type, intervention protocol, and study size. Taken together, the findings suggest that treatment choice involves trade-offs among efficacy, tolerability, procedural efficiency, and feasibility. Autolytic and enzymatic approaches may be preferred when pain control is a priority, whereas biological debridement may be considered when wound-bed cleansing or healing response is the main concern. Since the evidence remains heterogeneous and partly imprecise, treatment decisions should be guided by wound etiology, vascular status, infection burden, necrotic tissue burden, patient preference, and local resources.

Surgical debridement can rapidly and thoroughly remove necrotic tissue, but it requires considerable technical expertise and may cause unintended damage to viable tissue (11). Autolytic debridement promotes endogenous enzymatic activity by maintaining a moist environment. This method is relatively gentle and preserves the healthy tissue surrounding the wound, but acts slowly and is not suitable for wounds with severe infection or extensive necrosis (12). Enzymatic debridement uses exogenous proteolytic enzymes to degrade necrotic tissue and provides a degree of selectivity, although it is relatively costly and often combined with other methods to improve effectiveness (13). In recent years, physical debridement techniques have continued to develop. Ultrasonic debridement disrupts bacterial biofilms through cavitation effects, while waterjet debridement removes necrotic tissue using high-pressure fluid streams. Although these techniques can improve efficiency, their use is limited by the need for specialized equipment and higher costs (14).

Biological debridement, particularly maggot therapy, has attracted increasing attention because of its unique advantages. It is mainly indicated for chronic infected wounds that do not respond to conventional treatments. Previous studies have shown that this therapy accelerates debridement, shortens healing time, improves clinical outcomes, and reduces hospital stay compared with conventional dressings (15, 16). It is also suitable for patients with multiple comorbidities who are not candidates for surgical intervention, including pregnant women (17). The therapeutic effects of maggot therapy are mediated through both mechanical and biochemical mechanisms. Maggots physically ingest necrotic tissue, while their secretions contain proteolytic enzymes that degrade devitalized tissue and facilitate wound cleaning (18). Chronic infected wounds are often associated with mixed bacterial infections, with methicillin-resistant *Staphylococcus aureus* being a common pathogen (19). Antimicrobial peptides in maggot secretions inhibit such pathogens and contribute to infection control (20). In addition, enzymatic components in maggot secretions promote degradation of extracellular matrix proteins and support tissue remodeling (21, 22). Experimental studies have also shown that these secretions can activate signaling pathways related to wound healing, enhance extracellular matrix reconstruction, promote angiogenesis, and facilitate cell migration and proliferation (23). Furthermore, bioactive compounds derived from larvae have been reported to upregulate vascular endothelial growth factor expression, thereby enhancing neovascularization (24). Overall, maggot therapy exerts its effects through multiple coordinated mechanisms, including mechanical debridement, enzymatic activity, antimicrobial action, and modulation of cellular processes, making it a valuable option for difficult-to-heal wounds. However, its clinical use remains limited by patient acceptance. Surveys have indicated that approximately 36% of patients are willing to consider this therapy, and psychological aversion to maggots remains a significant barrier, particularly among female patients (10, 25). These findings highlight the importance of patient education and improved communication to increase acceptance. A major strength of this study is the application of a Bayesian network meta-analysis, which integrates evidence across multiple interventions within a single framework. This approach enables both direct and indirect comparisons and provides a comprehensive assessment of relative effectiveness. The inclusion of only randomized controlled trials enhances the reliability of the findings. In addition, multiple clinically relevant outcomes were evaluated, including wound healing, complete debridement, pain, procedure duration, and healing time, thereby improving the applicability of the results to clinical decision-making.

However, several limitations should be considered. First, outcome definitions varied across trials. Wound healing and complete debridement were extracted according to the definitions used in the original studies, but differences in follow-up duration, measurement criteria, and clinical endpoints may have contributed to heterogeneity and may have influenced pooled estimates. Second, important clinical effect modifiers, including wound etiology, baseline wound size and duration, infection status, necrotic or slough burden, vascular status, co-interventions, and background standard care, were not consistently reported across studies. These differences may have affected the transitivity assumption and, therefore, the interpretation of indirect comparisons. Third, the number of studies was limited for several outcomes, resulting in sparse evidence networks, imprecise estimates, and lower GRADE certainty for some comparisons. Fourth, standard care and conventional comparator arms were combined into a single SC node to maintain network connectivity, although their clinical components varied across trials. Similarly, some intervention classes included related but not identical protocols or products, which may have diluted class-specific effects. Fifth, the majority of the included trials evaluated single debridement strategies, whereas combined or sequential approaches are frequently used in clinical practice. Therefore, the ranking results should be interpreted as exploratory probability-based estimates rather than as evidence of clinically decisive superiority, and treatment selection should remain individualized according to wound etiology, vascular status, infection burden, necrotic tissue burden, patient tolerance, and resource availability.

## 6 Conclusion

The comparative performance of debridement strategies varied across outcomes in chronic lower-extremity wounds. Biological and enzymatic debridement ranked favorably for wound healing and complete debridement, whereas autolytic and enzymatic debridement were associated with lower pain scores. Ultrasound-assisted debridement may offer procedural efficiency advantages. However, no single approach was consistently optimal, and the findings should be interpreted in light of clinical heterogeneity, variable comparator treatments, sparse evidence, and uncertainty in several direct and indirect comparisons. Future trials should evaluate not only single debridement modalities but also combined or sequential strategies that better reflect real-world wound-bed preparation.

## Data Availability

The original contributions presented in the study are included in the article/Supplementary material, further inquiries can be directed to the corresponding author.
