# Supplementary material for: Comparative effectiveness of debridement strategies for chronic lower-extremity wounds: a systematic review and network meta-analysis
Source: Front Med (Lausanne). 2026 May 21;13:1852029. doi: 10.3389/fmed.2026.1852029 (PMC13233243; doi:10.3389/fmed.2026.1852029)
Supplement: Supplementary file 1 [file Supplementary_file_1.DOCX]

**Supplementary documents**

**Supplementary Table1:**Simplified transitivity assessment

**Supplementary Table 2.** PSRF/Rhat values for convergence assessment of Bayesian network meta-analysis models

**Supplementary Table3:**Summary of Findings and Quality of Evidence (GRADE)

**Supplementary Figure1:** Convergence diagnostics for Wound Healing.

**Supplementary Figure2:** League table of pairwise comparisons for Wound Healing gain from the network meta-analysis.

**Supplementary Figure3:** League table of pairwise comparisons for Complete Debridement gain from the network meta-analysis.

**Supplementary Figure4:** League table of pairwise comparisons for Pain Score gain from the network meta-analysis.

**Supplementary Figure5:** League table of pairwise comparisons for Procedure Time gain from the network meta-analysis.

**Supplementary Figure6:** League table of pairwise comparisons for Time to Healing gain from the network meta-analysis.

**Supplementary Figure7:** Node-splitting analysis assessing the consistency of the network meta-analysis for Wound Healing.

**Supplementary Figure8:** Heterogeneity test of Wound Healing.

**Supplementary Figure9:** Heterogeneity test of Complete Debridement.

**Supplementary Figure10.**Subgroup analyses based on conventional pairwise meta-analysis of wound healing

**Supplementary Table1.** Simplified transitivity assessment

| **Author** | **Baseline wound size/severity** | **Wound duration** | **Infection status** | **Necrotic/slough burden** | **Vascular status** | **Follow-up duration** | **Treatment frequency/duration** | **Co-interventions** | **Risk of bias** | **Transitivity concern** |
| --- | --- | --- | --- | --- | --- | --- | --- | --- | --- | --- |
| Saranholi | VLU; area 14.3 vs 10.3 cm2; PUSH 13.5 | 42 vs 24 months | Superficial infection included; deep infection excluded | Debris/necrosis by NERDS; baseline curettage | PAD excluded; ABI 1.05 vs 1.07 | 30 days | HAg ~q4d; collagenase daily | Compression in both groups | Low/some concerns | Moderate |
| Shoham (EX-02 vs gel) | VLU; 13.1 vs 18.8 cm2; NVT 72% vs 78% | 26.8 vs 39.5 weeks | Clinically significant infection excluded | >=50% necrotic/slough/fibrin required | Arterial insufficiency excluded | Up to 17 weeks | Up to 8 daily 24-h applications | Standard compression; active debridement prohibited | Low | Low to moderate |
| Shoham (EX-02 vs NSSOC) | VLU; 13.1 vs 14.4 cm2; NVT 72% vs 68% | 26.8 vs 26.1 weeks | Clinically significant infection excluded | >=50% necrotic/slough/fibrin required | Arterial insufficiency excluded | Up to 17 weeks | EX-02 up to 8 applications; NSSOC per label | Standard compression; active debridement prohibited | Low/some concerns | Moderate |
| Cangel | PAD ischemic wounds; Fontaine III-IV; size NR | NR | Infected/gangrenous wounds described; grading NR | Necrotic/gangrenous tissue; amount NR | All had PAD and revascularization; ABI ~0.45 | 1 month | VAC ~15 sessions; MDT ~6 sessions | Revascularization in all; debridement as needed | Some concerns | Moderate |
| Shoham (ESX vs gel) | Mixed lower-extremity ulcers; 33.6 vs 25.8 cm2; >=50% NVT | >=4 weeks; 73 vs 31 weeks | Deep/systemic infection excluded | >=50% non-viable tissue required | ABI <=0.7 or low flow excluded | Up to 6 months | Daily 4-h applications, up to 10 | Standard care at investigator discretion | Some concerns | Moderate |
| Alvarez | VLU; area 11.23 vs 9.89 cm2 | 8 vs 4 months | NR | Debridement required; amount NR | ABI >0.7 required | 24 weeks | Debridement as needed; US vs sharp | Same dressing and multilayer compression | Some concerns | Low to moderate |
| Colenci | VLU; median area 5.08 vs 5.71 cm2 | 12 vs 36 months | Infected/critical colonization excluded | Necrosis excluded; devitalized tissue assessed | PAD excluded; ABI ~1.0 | 90 days | Daily biomembrane or collagenase | Daily compression in both groups | Some concerns | Low to moderate |
| Michailidis | DFU; >=1 cm2; UT A/B 1-3; n=14 ulcers | >=1 month; exact NR | Managed infection allowed; gangrene excluded | Non-viable tissue removed; amount NR | Adequate pulses/toe pressure required | 6 months or healed | Weekly LFUD or NSSD | Dressings/offloading not standardized | Some concerns | Moderate |
| de Araujo (fibrin) | VLU; >=5 cm2; baseline similar | 62.9 vs 63.9 months | Severe infection excluded | Devitalized tissue required | Arterial/mixed ulcers excluded if ABI <0.9 | 60 days | Fibrin gel vs carbopol; visits q15d | Compression in all groups | Some concerns | Low to moderate |
| de Araujo (papain) | VLU; >=5 cm2; baseline similar | 57.9 vs 63.9 months | Severe infection excluded | Devitalized tissue required | Arterial/mixed ulcers excluded if ABI <0.9 | 60 days | 8% papain vs carbopol; visits q15d | Compression in all groups | Some concerns | Low to moderate |
| Liu | Delayed-healing wounds; 15.6 vs 19.6 cm2; depth 20 mm; NVT ~50% | >=30 days; by arm NR | Bacterial counts assessed | Devitalized tissue ~50% | ABI 1.1 vs 1.0; ischemic wounds excluded | 3-6 weeks post-closure | Operating-room hydrosurgery or conventional excision | Closure by primary intention/graft/flap | Some concerns | High |
| Rodrigues | VLU; 28 ulcers; baseline area NR | Mostly >10 years | Infected ulcers excluded | Devitalized tissue assessed; baseline amount NR | Non-palpable distal pulse excluded | 12 weeks | 2% papain or CMC; daily dressing | Dressing kit; compression NR | Some concerns | Low to moderate |
| Davies | VLU; area 10.8 vs 8.1 cm2; >=20% slough | New/recurrent; duration NR | Baseline infection NR | >=20% slough required | Venous reflux; ABPI 0.85-1.25 | 12 weeks | One larval dressing + 4LB vs 4LB alone | Multilayer compression in both groups | Some concerns | Low to moderate |
| Humbert | VLU; 31.0 vs 26.1 cm2; >70% fibrin/necrosis | 1.7 vs 1.5 years | Infection not fully reported | >70% fibrin/necrosis required | ABI >0.8; mean ~1.07 | 14 days | PA hydrogel daily; amorphous gel q24-72h | Compression in all patients | Some concerns | Low to moderate |
| Mudge | VLU/MLU; >=2 cm2; >25% slough/necrosis | 7 vs 8 months | Withdrawals often infection/increased slough | >25% slough/necrosis required | ABPI >=0.5; arterial-only excluded | Up to 21 days + final visit | Larvae or hydrogel q3-4d | Compression with Profore/Light | Some concerns | Moderate |
| Tallis | Neuropathic DFU; 0.5-10 cm2; mean 2.7 cm2 | >=1 month | Active infection excluded | Necrotic tissue scored; baseline sharp debridement | Adequate perfusion required | 12 weeks | CCO daily vs SMG daily +/- sharp debridement | Off-loading and standard dressings | Some concerns | Moderate |
| Opletalova | Venous lower-limb wounds; <=40 cm2; <2 cm deep | Non-healing; exact NR | Swabs for MRSA/Pseudomonas | Sloughing wound required | ABI >=0.8; neuropathic foot ulcers excluded | 30 days | MDT twice weekly vs sharp debridement 3x/week | No compression during 2-week hospitalization | Some concerns | Moderate to high |
| Herberger | Vascular leg ulcers; size NR | NR | Infection requiring treatment excluded | Necrosis/fibrin scored | Vascular origin; ABI NR | 4-12 days | Three UAW or surgical debridements | Pain therapy as needed | Some concerns | Moderate |
| Dumville | VLU/mixed ulcers; >=25% slough/necrosis | NR | Bacterial load/MRSA assessed | >=25% slough/necrosis required | ABI >=0.6 | Up to 12 months | Larvae q3-4d or hydrogel per nurse | Compression encouraged; withheld during larvae | Low/some concerns | Moderate |
| Caputo | DFU/VLU; area 5.9 vs 3.9 cm2; devitalized 5.3 vs 3.7 cm2 | 1.2 months both | Clinical infection excluded | Black/yellow devitalized tissue recorded | PVD details/ABI NR | 12 weeks | Single OR Versajet vs scalpel + pulsed lavage | Dressings; compression for VLU; offloading for DFU | Some concerns | High |
| Koenig | Chronic leg ulcers; size NR | NR | NR | Eschar/slough assessed | Venous insufficiency; ABI NR | 3 weeks | TenderWet or Iruxol N daily | Short-stretch compression in both groups | Some concerns | Low to moderate |
| Wayman | VLU; size 16 vs 18 cm2; slough 95% vs 100% | 4 vs 5 months | NR | Necrotic/sloughy ulcers required | Arterial insufficiency excluded | 1 month or debridement | Hydrogel or larvae up to 72 h/application | Leg ulcer service care; other co-care NR | Some concerns | Moderate |
| Westerhof | Necrotic VLU; 31 ulcers; size NR | 6 vs 4 months | NR; erythema/edema monitored | Necrotic/fibrinous tissue quantified | Arterial insufficiency excluded | 1 week initial assessment | Krill enzyme twice daily vs pregrafting regimen | Pregrafting pathway; compression/control care | Some concerns | Moderate |
| Lazaro-Martinez | Infected DFU; UT IB/IIB/ID/IID; 1-30 cm2 | 1-24 months | Mild/moderate infection included | Necrotic/devitalized tissue removed | Critical ischemia excluded | 6 weeks + 6 months | Weekly UAW or surgical debridement | Off-loading, moist dressings, antibiotics if needed | Some concerns | Moderate |
| Rastogi | DFU Wagner 2-3; 11.3 vs 14.8 cm2 | 15.8 vs 12.1 weeks | Infected or noninfected DFU; cultures/antibiotics if needed | Necrotic burden NR | ABI >=0.5 required | 28 days | Noncontact US/sham daily then twice weekly | Standard DFU care and offloading | Low/some concerns | Moderate |
| Zu | DFU; n=120/120; baseline size NR | Wound duration NR | Cultures and anti-infective therapy used | Necrotic tissue removed if large; amount NR | NR | 3 months | Traditional debridement vs ultrasonic daily/qod | Routine diabetes/wound care in both groups | Some concerns | Moderate |
| Cao | DFU Wagner <=3; area 11.5 vs 12.0 cm2 | 19.1 vs 19.2 days | Bacterial burden assessed | Necrotic burden NR | ABI ~0.99; ABI <0.6 excluded | 20 days | Standard care vs daily low-frequency US | Standard DFU care and dressings | Some concerns | Moderate |

**Supplementary Table 2.** PSRF/Rhat values for convergence assessment of Bayesian network meta-analysis models

| **Outcome** | **Model parameter** | **PSRF/Rhat** | **Upper CI** | **Convergence judgement** |
| --- | --- | --- | --- | --- |
| Wound healing | d.ENZ.MECH | 1.0000 | 1.0002 | Adequate |
| Wound healing | d.SC.AUTO | 1.0054 | 1.0142 | Adequate |
| Wound healing | d.SC.BIO | 1.0001 | 1.0002 | Adequate |
| Wound healing | d.SC.ENZ | 1.0063 | 1.0161 | Adequate |
| Wound healing | d.SC.US | 1.0005 | 1.0015 | Adequate |
| Wound healing | d.SURG.HYDRO | 1.0002 | 1.0004 | Adequate |
| Wound healing | d.US.SURG | 1.0012 | 1.0032 | Adequate |
| Complete debridement | d.AUTO.BIO | 1.0017 | 1.0052 | Adequate |
| Complete debridement | d.AUTO.ENZ | 1.0024 | 1.0070 | Adequate |
| Complete debridement | d.AUTO.SC | 1.0001 | 1.0003 | Adequate |
| Pain score | d.AUTO.ENZ | 1.0002 | 1.0007 | Adequate |
| Pain score | d.AUTO.SC | 1.0002 | 1.0003 | Adequate |
| Pain score | d.BIO.AUTO | 1.0000 | 1.0001 | Adequate |
| Pain score | d.BIO.SURG | 1.0001 | 1.0002 | Adequate |
| Pain score | d.SURG.US | 1.0001 | 1.0002 | Adequate |
| Procedure time | d.BIO.AUTO | 1.0007 | 1.0019 | Adequate |
| Procedure time | d.BIO.SURG | 1.0001 | 1.0003 | Adequate |
| Procedure time | d.SURG.US | 1.0004 | 1.0012 | Adequate |
| Time to healing | d.BIO.AUTO | 1.0002 | 1.0004 | Adequate |
| Time to healing | d.BIO.SURG | 1.0000 | 1.0002 | Adequate |
| Time to healing | d.SURG.US | 1.0005 | 1.0015 | Adequate |

Abbreviations: PSRF, potential scale reduction factor; CI, confidence interval; AUTO, autolytic debridement; BIO, biological debridement; ENZ, enzymatic debridement; HYDRO, hydrosurgical debridement; MECH, mechanical debridement; SC, standard care; SURG, surgical/sharp debridement; US, ultrasound-assisted debridement.PSRF/Rhat values close to 1.00 indicate adequate convergence. The table combines convergence diagnostics across all evaluated outcomes.

**Supplementary Table3:**Summary of Findings and Quality of Evidence (GRADE)

| **Quality assessment** | | | | | | | **Summary of Findings** | | | | |
| --- | --- | --- | --- | --- | --- | --- | --- | --- | --- | --- | --- |
| **Participants (studies) Follow up** | **Risk of bias** | **Inconsistency** | **Indirectness** | **Imprecision** | **Publication bias** | **Overall quality of evidence** | **Study event rates (%)** | | **Relative effect** (95% CI) | **Anticipated absolute effects** | |
|  |  |  |  |  |  |  | **With Control** | **With Outcomes** |  | **Risk with Control** | **Risk difference with Outcomes** (95% CI) |
| **Wound Healing** (CRITICAL OUTCOME) | | | | | | | | | | | |
| 1379 (18 studies) | no serious risk of bias | no serious inconsistency | no serious indirectness | serious | undetected | ⊕⊕⊕⊝ **MODERATE** due to imprecision | 203/548  (37.0%) | 303/580  (52.2%) | **OR 1.86**  (1.47 to 2.36) | **Study population** | |
|  |  |  |  |  |  |  |  |  |  | **370 per 1000** | **152 more per 1000**  (93 to 211 more) |
|  |  |  |  |  |  |  |  |  |  | **Moderate** | |
|  |  |  |  |  |  |  |  |  |  | **558 per 1000** | **72 more per 1000** (from 85 fewer to 206 more) |
| **Complete Debridement** (CRITICAL OUTCOME) | | | | | | | | | | | |
| 408 (5 studies) | no serious risk of bias | no serious inconsistency | no serious indirectness | serious | undetected | ⊕⊕⊕⊝ **MODERATE** due to imprecision | 46/158  (29.1%) | 96/177  (54.2%) | **OR 2.89**  (1.83 to 4.54) | **Study population** | |
|  |  |  |  |  |  |  |  |  |  | **291 per 1000** | **251 more per 1000**(139 to 360 more) |
|  |  |  |  |  |  |  |  |  |  | **Moderate** | |
|  |  |  |  |  |  |  |  |  |  | **302 per 1000** | **292 more per 1000** (from 189 more to 387 more) |
| **Pain Score** (CRITICAL OUTCOME; Better indicated by lower values) | | | | | | | | | | | |
| 422 (5 studies) | serious | serious | no serious indirectness | no serious imprecision | undetected | ⊕⊕⊝⊝ **LOW** due to risk of bias, inconsistency | 215 | 207 | **-** |  | The mean pain score in the intervention groups was **0.31 higher** (0.19 lower to 0.8 higher) |
| **Procedure Time** (CRITICAL OUTCOME; Better indicated by lower values) | | | | | | | | | | | |
| 281 (4 studies) | no serious risk of bias | no serious inconsistency | serious | no serious imprecision | undetected | ⊕⊕⊕⊝ **MODERATE** due to indirectness | 142 | 139 | **-** |  | The mean procedure time in the intervention groups was **2.6 lower** (3.19 to 2.01 lower) |
| **Time to Healing** (CRITICAL OUTCOME; Better indicated by lower values) | | | | | | | | | | | |
| 586 (7 studies) | no serious risk of bias | serious | no serious indirectness | serious | undetected | ⊕⊕⊝⊝ **LOW** due to inconsistency, imprecision | 293 | 293 | **-** |  | The mean time to healing in the intervention groups was **7.34 lower** (7.84 to 6.84 lower) |

**Supplementary Figure1:** Convergence diagnostics for Wound Healing.

**Supplementary Figure2:** League table of pairwise comparisons for Wound Healing gain from the network meta-analysis.

**Supplementary Figure3:** League table of pairwise comparisons for Complete Debridement gain from the network meta-analysis.

**Supplementary Figure4:** League table of pairwise comparisons for Pain Score gain from the network meta-analysis.

**Supplementary Figure5:** League table of pairwise comparisons for Procedure Time gain from the network meta-analysis.

**Supplementary Figure6:** League table of pairwise comparisons for Time to Healing gain from the network meta-analysis.

**Supplementary Figure7:** Node-splitting analysis assessing the consistency of the network meta-analysis for Wound Healing .

**Supplementary Figure8:** Heterogeneity test of Wound Healing.

**Supplementary Figure9:** Heterogeneity test of Complete Debridement.

**Supplementary Figure10.** Subgroup analyses based on conventional pairwise meta-analysis of wound healing.**(A)**Subgroup analysis according to wound etiology.**(B)** Subgroup analysis according to debridement strategy.
